# The correlation between dietary inflammatory index and risk of hyperuricemia in the U.S. population

**DOI:** 10.1097/MD.0000000000033374

**Published:** 2023-05-17

**Authors:** Lijuan Wang, Huoliang Liu, Dan Wang, Xiaoyan Huang, Xiaofan Hong, Yi Wang, Ping Li, Kun Bao, Daixin Zhao

**Affiliations:** a Second Clinical Medical College, Guangzhou University of Chinese Medicine, Guangzhou, China; b The Affiliated TCM Hospital of Guangzhou Medical University, Guangzhou, China; c Department of Nephrology, Guangdong Provincial Hospital of Chinese Medicine, Guangzhou, China; d State Key Laboratory of Dampness Syndrome of Chinese Medicine, The Second Affiliated Hospital of Guangzhou University of Chinese Medicine, Guangzhou, China.

**Keywords:** cross-sectional study_4_, dietary inflammatory index_1_, hyperuricemia_3_, national health and nutrition examination survey (NHANES)_5_, serum uric acid_2_

## Abstract

The dietary inflammatory index (DII) has been reported to be related to chronic diseases as a novel inflammatory marker. However, the correlation between DII score and hyperuricemia in adults in the United States is still unclear. Therefore, our goal was to explore the correlation between them. A total of 19,004 adults were enrolled in the National Health and Nutrition Examination Survey from 2011 to 2018. DII score was calculated according to 28 dietary items obtained by 24-hour dietary interview data. Hyperuricemia was defined by serum uric acid level. We used multilevel logistic regression models and subgroup analysis to determine whether the 2 were associated. DII scores were positively associated with serum uric acid and the risk of hyperuricemia. Per unit increased in DII score was associated with a 3 mmol/L increase in serum uric acid in males (β 3.00, 95% confidence interval (CI) 2.05–3.94) and 0.92mmol/L in females (β 0.92, 95% CI 0.07–1.77), respectively. Compared with the lowest tertile of DII score, the rise of DII grade increased the risk of hyperuricemia among the whole participants (*T*2: odds ratio (OR) 1.14, 95% CI 1.03, 1.27; *T*3: OR 1.20 [1.07, 1.34], *P* for trend = .0012) and males [*T*2: 1.15 (0.99, 1.33), *T*3: 1.29 (1.11, 1.50), *P* for trend = .0008]. For females, the correlation between DII score and hyperuricemia was statistically significant in the subgroup stratified by body mass index (BMI) (BMI < 30, OR 1.08, 95% CI 1.02–1.14, *P* for interaction = .0134), which indicates that the association depends on BMI. In the United States male population, the DII score has a positive correlation with hyperuricemia. Anti-inflammatory dietary intake can be beneficial for lower serum uric acid.

## 1. Introduction

An estimated 15% to 20% of the global population suffers from hyperuricemia (HUA).^[1]^ HUA is defined by serum uric acid concentration, resulting predominantly from purine metabolism disorders or uric acid excretion disorders.^[2]^ It links to higher risks of gout,^[3]^ kidney diseases,^[4]^ diabetes mellitus,^[5]^ and cardiovascular disease.^[6,7]^ HUA, as a burden of public healthcare on society globally, accounts for tremendous medical costs.^[8]^

The risk factors of HUA have been investigated and are not yet fully clear. Recently, epidemiologic and clinical data have shown what contributes to HUA, including genetic factors,^[9]^ obesity,^[10]^ environment,^[11]^ diuretics use,^[12,13]^ and diet,^[14–16]^ and alterations in dietary patterns may have an influence on the HUA endpoints.^[15,17]^ A dietary approaches to stop hypertension diet^[18]^ and Mediterranean diet^[19]^ can lower the level of serum uric acid, while a western diet is linked to a higher risk of HUA.^[20]^ Furthermore, previous research suggests dietary patterns play a role in regulating inflammation. Plant-based dietary patterns are closely correlated with a low level of inflammation and oxidative stress.^[21]^ The research community is growing increasingly concerned about the effect of diet inflammatory modulation in HUA for prevention and treatment.^[22]^

The dietary inflammatory index (DII) is an innovative dietary tool based on literature to measure the inflammatory potential of diet in all the population.^[23]^ It is based on the pro-inflammatory and anti-inflammatory properties of 45 food parameters.^[24]^ DII scores are regulated to global dietary intakes, and different cultures and their dietary patterns can benefit from their use.

Higher DII scores indicate a pro-inflammatory diet, while lower DII scores indicate an anti-inflammatory diet. Diet can modulate the inflammatory level via pro- or anti-inflammatory mechanisms.^[25]^ Chronic inflammation is involved in the pathogenesis of HUA.^[26]^

However, the association between the consumption of a pro-inflammatory dietary and HUA has not been explored using a nationally representative sample in the United States before. We assumed that the higher dietary inflammatory index was correlated with increased risk of HUA in this cross-sectional study.

## 2. Materials and Methods

### 2.1. Data source and study population

The National Health and Nutritional Examination Survey (NHANES) is a nationwide cross-sectional survey with a multistage probability sample method to assess the health and nutrition state of the civilian in the United States updated every 2 years by interviews, physical examination and laboratory tests. The NHANES has been approved by the National Center for Health Statistics Ethics Review Board. All study participants signed informed consent. The detailed information is available on a site (www.cdc.gov/nchs/nhanes/).

**Figure 1.**
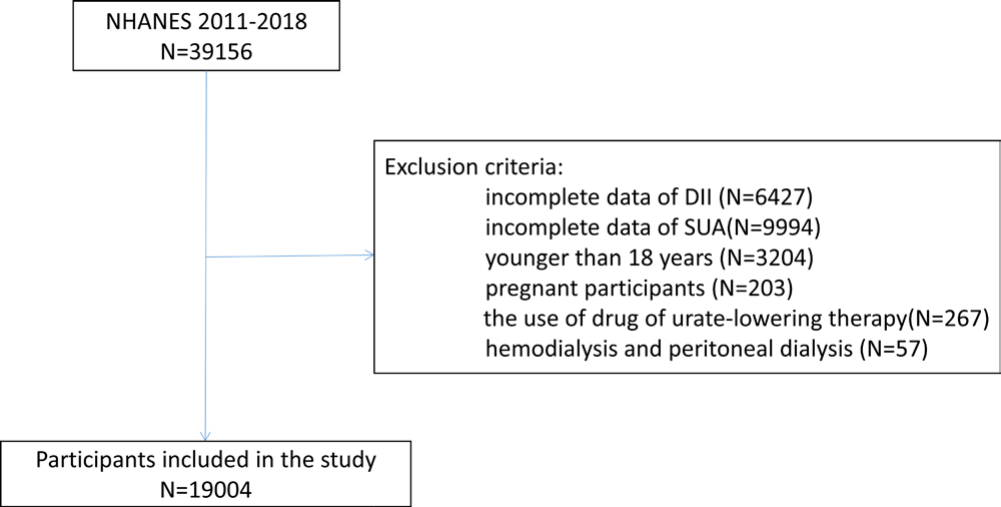
Participants included in the study.

We obtained the data from NHANES 2011 to 2018. The exclusion criteria for participants in this study were: Incomplete date of DII and serum uric acid; Younger than 18 years and pregnant women; The use of drug of urate-lowering therapy; Individuals with hemodialysis and peritoneal dialysis. In total, the sample size was 19,004 (Fig. 1).

### 2.2. DII

We collected dietary intakes from the first 24-hour dietary recall interview conducted in person at the mobile examination center, which covered total nutrient intake for the first 24 hours. NHANES Dietary Interviewers Procedure Manuals provide detailed descriptions of dietary interview techniques. The DII, a literature-derived dietary index, was developed to estimate the inflammatory of populations diet potential.^[24]^ Inflammatory biomarkers (IL-1β, IL-4, IL-6, IL-10, TNF-α, and C-reactive protein) were determined to assess the effect of food on inflammation. The pro-inflammatory food parameter is labeled in “+,” the anti-inflammatory parameter is labeled in “−” and the neutral parameter without any effect on inflammatory is labeled in “0.” DII scores in this study were calculated by 28 of 45 food parameters, including energy, carbohydrates, protein, alcohol, fiber, cholesterol, total fat, saturated fat, monounsaturated fatty acid, polyunsaturated fatty acid, n-3 fatty acids, n-6 fatty acids, niacin, vitamin A, vitamin B1, vitamin B2, vitamin B6, vitamin B12, vitamin C, vitamin D, vitamin E, iron, magnesium, zinc, selenium, folic acid, beta carotene, and caffeine. The dietary inflammation index was calculated based on dietary intake data from a regionally representative world database. First, subtracting the individual’s intake from the standard global mean to created z-score, and divided it by standard deviation. Secondly, this value is converted to a symmetrical distribution which is centered at zero, between −1 and 1. Thirdly, multiply that by the inflammatory effect score for each food and sum all food parameter DII values to create an overall DII score.

### 2.3. Definition of HUA

HUA was defined as ≥ 7 mg/dL (416.4 mmol/L) for males, or ≥ 6 mg/dL (356.9 mmol/L) for females.^[27]^

### 2.4. Assessment of other covariates

The covariates included: age (< 60 years and ≥ 60 years), gender (female and male), race (non-Hispanic White, non-Hispanic Black, Mexican American, or other race), body mass index (< 30 and ≥ 30 kg/m2), family income-to-poverty ratio (< 1.3, 1.3–3.5, ≥ 3.5), education background (less than high school, high school or equivalent and college or above), hypertension, diabetes, estimated glomerular filtration rate (eGFR), smoking status, alcohol intake, and energy intake. Smoking status and alcohol intake were divided into 3 groups: never, former and current. Energy intake was categorized as < 500 kcal, 500–4000 kcal and ≥ 4000 kcal, which corresponded to deficiency of intake, normal, and excessive energy intake participants, respectively. Except for eGFR, the details of the acquisition process as well as the determination of each variable are available at the following Web site: www.cdc.gov/nchs/nhanes. eGFR was measured applying the Chronic Kidney Disease Epidemiology Collaboration equation and was categorized as < 60 and ≥ 60 mL/minutes/1.73m2.^[28]^

### 2.5. Statistical analysis

Continuous variables were present as mean ± SD, and categorical variables were expressed as frequency and percentage. Student *t* test was used to analyze continuous variables. To examine categorical variables, the chi-square test was applied. Multivariate linear regression models were set up to assess the correlation between DII score, serum uric acid and HUA. Stratified analyses were carried out to evaluating the interaction effect. Dietary intakes were shown according to HUA status. We completed all analyses using R (Version 3.4.3) (http://www.R-project.org, The R Foundation) and Empower Stats software (http://www.empowerstats.com). In all analyses, a value of *P* < .05 (2-sided) was considered to indicate statistical significance.

## 3. Results

This study involved a total of 19,004 individuals (48.83% males vs 51.17% females). The prevalence of HUA was 19.83% and it was higher in males than in females (22.45% vs 17.33%). The participant with HUA was more likely to be male, old, non-Hispanic, hypertensive, diabetes, chronic kidney disease, and have higher BMI levels (*P* < .05). DII scores ranged from −5.03 to 5.79. Compared with the non-HUA group, participants in the HUA group had higher DII scores (*P* < .001). The details of the study participants characteristics are shown in Table 1.

**Table 1:** Baseline characteristics of the participants according to gender and hyperuricemia status.

Characteristics	Female				Male			
	Total	Non-Hyperuricemia	Hyperuricemia	*P* value	Total	Non-Hyperuricemia	Hyperuricemia	*P* value
	(n = 9725)	(n = 8040)	(n = 1685)		(n = 9279)	(n = 7196)	(n = 2083)	
SUA (mmol/L)	289.00 ± 77.20	262.83 ± 50.10	413.88 ± 59.67	<.001	359.66 ± 77.87	328.52 ± 52.65	467.25 ± 50.13	<.001
DII	1.84 ± 1.84	1.80 ± 1.86	2.02 ± 1.77	<.001	1.14 ± 1.91	1.08 ± 1.91	1.36 ± 1.89	<.001
Age (yr)				<.001				.002
<60	6703	5876 (73%)	827 (49%)		6346	4978 (69%)	1368 (66%)	
>=60	3022	2164 (27%)	858 (51%)	`	2933	2218 (31%)	715 (34%)	
Race (n, %)				<.001				<.001
Non-Hispanic white	3634	2944 (37%)	690 (41%)		3601	2771 (39%)	830 (40%)	
Non-Hispanic black	2185	1690 (21%)	495 (29%)		1983	1488 (21%)	495 (24%)	
Mexican American	1382	1223 (15%)	159 (9%)		1332	1103 (15%)	229 (11%)	
Other hispanic	1095	972 (12%)	123 (7%)		892	719 (10%)	173 (8%)	
Other race	1429	1211 (15%)	218 (13%)		1471	1115 (15%)	356 (17%)	
Family income-to-poverty ratio (n, %)				<.001				.01
**<**1.3	3051	2506 (34%)	545 (36%)		2637	2080 (32%)	557 (30%)	
1.3–3.5	3284	2666 (36%)	618 (41%)		3184	2495 (38%)	689 (37%)	
≥3.5	2521	2159 (29%)	362 (24%)		2622	1984 (30%)	638 (34%)	
Education background (n, %)				.967				.021
Less than high school	1979	1640 (20%)	339 (20%)		2153	1714 (24%)	439 (21%)	
High school or equivalent	2291	1893 (24%)	398 (24%)		2395	1859 (26%)	536 (26%)	
College or above	5449	4502 (56%)	947 (56%)		4723	3618 (50%)	1105 (53%)	
BMI (kg/m2)				<.001				<.001
<30	5544	4950 (62%)	594 (36%)		6020	5016 (70%)	1004 (49%)	
≥30	4086	3011 (38%)	1075 (64%)		3169	2120 (30%)	1049 (51%)	
Hypertension (n, %)				<.001				<.001
No	5732	5185 (64%)	547 (32%)		5409	4448 (62%)	961 (46%)	
Yes	3993	2855 (36%)	1138 (68%)		3870	2748 (38%)	1122 (54%)	
Diabetes (n, %)				<.001				.002
No	8004	6882 (86%)	1122 (67%)		7498	5864 (81%)	1634 (78%)	
Yes	1721	1158 (14%)	563 (33%)		1781	1332 (19%)	449 (22%)	
eGFR (mL/min/1.73m2)				<.001				<.001
<60	752	317 (4%)	435 (26%)		689	358 (5%)	331 (16%)	
≥60	8973	7723 (96%)	1250 (74%)		8590	6838 (95%)	1752 (84%)	
Smoking status (n, %)				<.001				<.001
Never	6450	5414 (68%)	1036 (62%)		4470	3447 (49%)	1023 (50%)	
Former	1651	1255 (16%)	396 (24%)		2589	1933 (27%)	656 (32%)	
Current	1496	1247 (16%)	249 (15%)		2087	1705 (24%)	382 (19%)	
Alcohol intake (n, %)				<.001				.685
Never	1871	1546 (22%)	325 (22%)		861	670 (10%)	191 (10%)	
Former	1060	803 (11%)	257 (17%)		1147	901 (14%)	246 (13%)	
Current	5765	4837 (67%)	928 (61%)		6503	5034 (76%)	1469 (77%)	
Energy intake (kcal)				.078				.004
<500	113	87 (1%)	26 (2%)		48	29 (1%)	19 (1%)	
500–4000	9466	7825 (97%)	1641 (97%)		8492	6575 (91%)	1917 (92%)	
≥4000	146	128 (2%)	18 (1%)		739	592 (8%)	147 (7%)	

DII = dietary inflammatory index, eGFR = estimated glomerular filtration rate.

The correlation between DII scores and serum uric acid examined by multivariate regression analysis is shown in Table 2. As the increase of DII scores, the serum uric acid tended to be higher. As continuous variable, per unit increased in DII score was associated with a 3 mmol/L increase in serum uric acid of male (β 3.00, 95% CI 2.05–3.94) and 0.92 mmol/L of female (β 0.92, 95% CI 0.07–1.77) adjusted for all covariates. We further converted DII score from a continuous variable to a categorical variable to conduct the sensitivity analysis. Compared with the lowest tertile of DII group (*T*1), the participants of the others have higher serum uric acid among males (*T*2: β 5.88, 95% CI 1.75–10.01; *T*3: β 12.74, 95% CI 8.41–17.08). However, this association is not significant among females (*T*2: β 3.12, 95% CI −0.54–6.79, *T*3: β 4.55, 95% CI 0.65–8.24).

**Table 2:** Association between DII and serum uric acid.

	Tertile of DII			*P* for trend	Continuous DII
	*T*1	*T*2	*T*3		
	Female				
Range of DII	−4.83 to 1.23	1.23 to 2.92	2.92–5.79		
Model 1	Reference	7.63 (3.88, 11.38) < 0.0001	11.84 (8.09, 15.59) < 0.0001	<.0001	2.69 (1.86, 3.52) < 0.0001
Model 2	Reference	5.92 (2.29, 9.55) 0.0014	10.30 (6.66, 13.94) < 0.0001	<.0001	2.35 (1.55, 3.16) < 0.0001
Model 3	Reference	3.12 (−0.54, 6.79) 0.0952	4.45 (0.65, 8.24) 0.0216	.0189	0.92 (0.07, 1.77) 0.0337
	Male				
Range of DII	−5.03 to 0.28	0.28 to 2.21	2.21–5.48		
Model 1	Reference	7.14 (3.27, 11.00) 0.0003	15.32 (11.46, 19.19) < 0.0001	<.0001	3.42 (2.59, 4.24) < 0.0001
Model 2	Reference	7.02 (3.16, 10.88) 0.0004	14.58 (10.69, 18.47) < 0.0001	<.0001	3.30 (2.47, 4.14) < 0.0001
Model 3	Reference	5.88 (1.75, 10.01) 0.0053	12.74 (8.41, 17.08) < 0.0001	<.0001	3.00 (2.05, 3.94) < 0.0001

DII = dietary inflammatory index.

We observed a positive association between DII score and HUA in Table 3. This association was significant among the whole participants (OR 1.05, 95% CI 1.02–1.07) and males (OR 1.06, 95% CI 1.03–1.10) after adjusting for all covariates, but not among females (OR 1.02, 95% CI 0.99–1.06). Compared with the lowest tertile of DII score, the rise of DII grade increased the risk of HUA among the whole participants (*T*2: OR 1.14, 95% CI 1.03, 1.27; *T*3: OR 1.20 [1.07, 1.34], *P* for trend = .0012) and males [*T*2: 1.15 (0.99, 1.33), *T*3: 1.29 (1.11, 1.50), *P* for trend = .0008].

**Table 3:** Association between DII and hyperuricemia.

	Tertile of DII			*P* for trend	Continuous DII
	*T*1	*T*2	*T*3		
	Overall participants				
Range of DII	−5.03 to 0.73	0.73–2.60	2.60–5.79		
Cases	1127/6335	1271/6334	1370/6335		
Model 1	Reference	1.16 (1.06, 1.27) 0.0011	1.28 (1.17, 1.39) < 0.0001	.0001	1.06 (1.04, 1.08) < 0.0001
Model 2	Reference	1.19 (1.09, 1.30) 0.0002	1.32 (1.20, 1.44) < 0.0001	.0001	1.07 (1.05, 1.09) < 0.0001
Model 3	Reference	1.14 (1.03, 1.27) 0.0151	1.20 (1.07, 1.34) 0.0013	.0012	1.05 (1.02, 1.07) 0.0004
	Female				
Range of DII	−4.83 to 1.23	1.23–2.92	2.92–5.79		
Cases	484/3242	574/3241	627/3242		
Model 1	Reference	1.23 (1.07, 1.40) 0.0025	1.37 (1.20, 1.56) < 0.0001	<.0001	1.07 (1.04, 1.10) < 0.0001
Model 2	Reference	1.18 (1.03, 1.35) 0.0192	1.32 (1.16, 1.51) < 0.0001	<.0001	1.06 (1.03, 1.10) < 0.0001
Model 3	Reference	1.10 (0.93, 1.29) 0.2529	1.11 (0.94, 1.31) 0.2158	.2015	1.02 (0.99, 1.06) 0.2381
	Male				
Range of DII	−5.03 to 0.28	0.28–2.21	2.21–5.48		
Cases	605/3093	687/3093	791/3093		
Model 1	Reference	1.17 (1.04, 1.33) 0.0104	1.41 (1.25, 1.59) < 0.0001	<.0001	1.08 (1.05, 1.11) < 0.0001
Model 2	Reference	1.17 (1.03, 1.32) 0.0135	1.38 (1.22, 1.55) < 0.0001	<.0001	1.08 (1.05, 1.10) < 0.0001
Model 3	Reference	1.15 (0.99, 1.33) 0.0612	1.29 (1.11, 1.50) 0.0008	.0008	1.06 (1.03, 1.10) 0.0002

DII = dietary inflammatory index.

**Figure 2.**
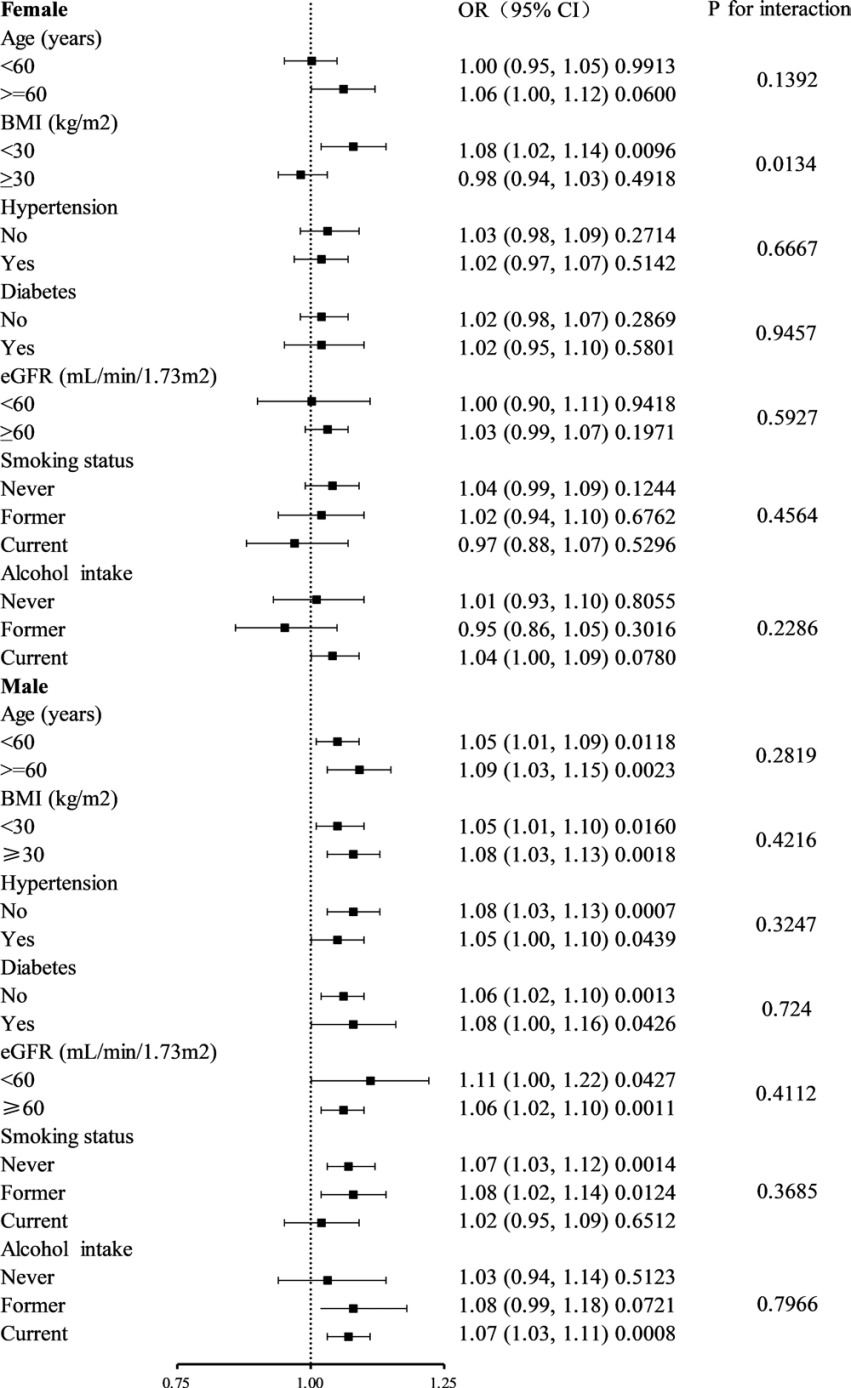
Subgroup analysis for the association between DII and hyperuricemia. DII = dietary inflammatory index.

Meanwhile, we conducted subgroup analysis to further explore the association between DII and HUA (Fig. 2). For females, there was a statistically significant difference in the subgroup stratified by BMI (BMI < 30, OR 1.08, 95% CI 1.02–1.14, *P* for interaction = .0134), indicating that the association depends on BMI. For males, the interaction test showed that there was no significant difference in subgroup stratified by age, BMI, hypertension, diabetes, eGFR, smoking status and alcohol intake (*P* for interaction > .05).

The results show that the subgroup analysis was adjusted for all presented covariates except the effect modifier.

The characteristics of dietary intakes stratified by HUA are shown in Table 4. To eliminate the effect of dietary energy, all dietary intakes were adjusted for total energy intake. Energy and carbohydrate intake in individuals with HUA are lower than those without HUA. People with HUA have higher intake of alcohol, cholesterol, niacin, vitamin D, and caffeine compared to participants without HUA. But it was significantly higher in fiber, saturated fat, vitamin A, vitamin B1, vitamin B2, vitamin C, vitamin E, iron, magnesium, zinc, folic acid, and caffeine of non-HUA.

**Table 4:** Characteristics of the dietary intakes (adjusted by energy, per 1000 kcal).

Dietary intakes	Non-hyperuricemia	Hyperuricemia	*P* value
Energy (kcal)	2137.22 ± 1006.70	2060.17 ± 991.10	<.001
Carbohydrate (g)	122.27 ± 27.95	118.57 ± 30.02	<.001
Protein (g)	39.30 ± 13.47	39.85 ± 14.39	.223
Alcohol (g)	3.53 ± 9.20	5.36 ± 12.33	<.001
Fiber (g)	8.57 ± 4.74	8.01 ± 4.66	<.001
Cholesterol (g)	142.59 ± 102.49	149.31 ± 113.74	<.001
Total fat (g)	38.00 ± 10.13	37.87 ± 10.94	.491
Saturated fat (g)	12.19 ± 4.41	11.94 ± 4.59	.002
MUFAs (g)	13.32 ± 4.37	13.29 ± 4.54	.734
PUFAs (g)	8.98 ± 3.90	9.12 ± 4.18	.284
Niacin (mg)	12.38 ± 6.39	12.65 ± 5.74	.018
Vitamin A (mcg RAE)	305.91 ± 334.42	303.02 ± 444.04	<.001
Vitamin B1 (mg)	0.78 ± 0.35	0.75 ± 0.33	<.001
Vitamin B2 (mg)	1.00 ± 0.56	0.96 ± 0.48	<.001
Vitamin B6 (mg)	1.04 ± 0.85	1.02 ± 0.66	.348
Vitamin B12 (mcg)	2.34 ± 2.72	2.39 ± 3.56	.273
Vitamin C (mg)	42.12 ± 48.94	40.77 ± 52.04	<.001
Vitamin D (mcg)	2.24 ± 2.77	2.29 ± 3.35	<.001
Vitamin E (mg)	4.23 ± 2.85	4.10 ± 2.66	.013
Iron (mg)	7.03 ± 3.49	6.81 ± 3.32	<.001
Magnesium (mg)	148.77 ± 60.71	144.91 ± 57.44	<.001
Zinc (mg)	5.28 ± 2.49	5.25 ± 3.57	<.001
Selenium (mg)	55.41 ± 22.86	56.62 ± 24.88	.082
Folic acid (mcg)	194.42 ± 107.40	185.59 ± 106.09	<.001
Beta carotene (mg)	1209.58 ± 2581.40	1240.41 ± 2725.09	.516
Caffeine (mg)	75.36 ± 137.17	77.85 ± 118.99	.008

## 4. Discwussion

We conducted cross-sectional analyses using NHANES data to explore the correlation between DII and HUA. Our finding showed that a higher DII score, indicative of a pro-inflammatory diet, was linked to a higher risk of HUA following adjustment for confounding factors. Compared with the lowest tertile of DII group, the highest tertile of DII group was associated with 20% increased risk of HUA in the whole participants, and 29% in males. For females, there was a significant correlation of BMI with DII score for risk of HUA (BMI < 30 vs BMI ≥ 30, *P* for interaction = .0134).

The correlations between dietary inflammation and HUA have been explored previously. In a recent study among a Chinese population, the DII scores had a highly positive association with the level of serum uric acid irrespective of gender.^[22]^ However, another interesting finding of a Korean study is that participants with a higher DII score have a higher risk of HUA in females only.^[29]^ Nevertheless, our study revealed this correlation was similar for both male and nonobese female subjects.

On the 1 hand, we propose that gap in dietary habits of different populations may contribute to this result. Korean traditional food is rich in fermented foods and seafood.^[30]^ The traditional Chinese diet contains a vast amount of cereals and vegetables and a small number of meat.^[31]^ The Western diet features in high fat consumption.^[32]^ In western countries, processed meats and red meats have already become the main component of the meat pattern.^[33]^ In China, the total meat intake largely is fresh pork.^[34]^

Pro-inflammatory dietary mainly contains red and processed meats, fried foods, high-sugar foods, and refined grains.^[35]^ While anti-inflammatory dietary contains more soy products, whole grains, nuts, vegetables, and fruits. Individuals with a more pro-inflammatory diet are more prone to HUA. This may be because a pro-inflammatory diet has higher purine content.^[31]^ The purine degradation leads to the formation of uric acid.^[36]^ And the enzyme xanthine oxidoreductase facilitates this degradation.^[37]^ Uric acid is excreted by the kidney.

On the other hand, previous studies have demonstrated estrogenic compounds could increase renal uric acid excretion.^[38]^ Levels of serum uric acid in females varies with the course of the menstrual cycle and higher levels of endogenous estradiol could result in suppression of uric acid.^[39]^ In the case of obesity, estrogen levels in the circulation increase by several pathways.^[40]^ Fat cells produce estrogen and aromatase activity increases estrogen levels as well.^[41]^ In addition, estrogen contributes to alleviating inflammation.^[25]^ Thus, for obesity alone, estrogen exerts their biological effects on uric acid far beyond dietary inflammation itself.

The higher the DII score, the more pro-inflammatory the diet. And inflammation has a close relationship with HUA as well as. Inflammation may elevate serum uric acid through multiple potential mechanisms, and oxidative stress is thought to have a key influence in the inflammatory response.^[42]^ The inflammatory response is a pathological characteristic of HUA.^[43]^ Uric acid acts as a danger signal and triggers inflammatory reactions.^[44]^ HUA has also been found to elicit an inflammatory process in kidneys.^[45]^ A recent study suggested anti-inflammatory diets represented by the Mediterranean diet^[21,46]^ enhance the plasma antioxidant potency^[21]^ and reduce xanthine oxidase activity,^[21]^ in turn, decreasing uric acid production. A recent study showed that parsley and celery have the potency to reduce inflammatory effects, increase antioxidant activities and improved renal dysfunction in hyperuricemic mice.^[47]^ However, further studies are still needed to explore the underlying mechanisms.

Yet, there were certain limitations in the current study. First, our study cannot draw a conclusion about causality for a retrospective, cross-sectional design. Secondly, serum uric acid was measured based on a single blood sample and dietary data are subject to recall bias, which may affect the accuracy of the results. Thirdly, the subjects with the risk of HUA had the potential to adjust their dietary habits. We adjusted for relevant variables wherever possible in our study, but serum uric acid is affected by many factors, such as daily water intake and metabolic factors.

## 5. Conclusions

Our study suggests that a higher DII score was linked to the higher risks of HUA in the United States adult population. Large-scale, prospective studies are needed to confirm our findings further.

## Author contributions

**Conceptualization:** Lijuan Wang.

**Data curation:** Xiaofan Hong.

**Funding acquisition:** Kun Bao.

**Methodology:** Huoliang Liu, Daixin Zhao.

**Resources:** Yi Wang, Ping Li, Xiaoyan Huang.

**Supervision:** Kun Bao, Daixin Zhao.

**Validation:** Lijuan Wang, Xiaofan Hong.

**Visualization:** Huoliang Liu, Dan Wang.

**Writing – original draft:** Lijuan Wang, Huoliang Liu, Dan Wang.

**Writing – review & editing:** Kun Bao, Daixin Zhao.
